# Fetal Weight Extrapolation following a Third-Trimester Ultrasound Examination Using the Gestation-Adjusted Projection Method: A Systematic Review and Meta-analysis

**DOI:** 10.1055/a-2628-2364

**Published:** 2025-06-24

**Authors:** Micah M. Vaughn-Valencia, Yan D. Zhao, Rodney K. Edwards, Shari Clifton, Hugh C. G. Nadeau

**Affiliations:** 1 Section of Maternal-Fetal Medicine, Department of Obstetrics and Gynecology, College of Medicine, University of Oklahoma Health Sciences Center, Oklahoma City, Oklahoma; 2 Department of Biostatistics and Epidemiology, Hudson College of Public Health, University of Oklahoma Health Sciences Center, Oklahoma City, Oklahoma; 3 Division of Maternal-Fetal Medicine, Department of Obstetrics and Gynecology, College of Medicine, University of Florida, Gainesville, Florida; 4 Department of Health Sciences Library and Information Management, Graduate College, University of Oklahoma Health Sciences Center, Oklahoma City, Oklahoma

**Keywords:** birth weight, estimated fetal weight, extrapolation, gestation-adjusted projection, meta-analysis, systematic review, ultrasound

## Abstract

**Objective:**

Using systematic review and meta-analysis methodology, we sought to evaluate the accuracy of the gestation-adjusted projection (GAP) method of fetal weight extrapolation in the prediction of actual birth weight.

**Study Design:**

A systematic literature search was performed using MEDLINE/PubMed, Embase, Scopus, and Web of Science for studies published from database inception to June 2023. Studies were compiled that assessed the accuracy of the GAP method in pregnant women at term (≥37 weeks gestation) with an ultrasound performed at 34 to 36 weeks gestation. Quality was assessed using the Newcastle–Ottawa scale, and risk of bias was assessed using the risk of bias in nonrandomized studies of interventions (ROBINS-I) tool. Meta-analysis was performed to evaluate the agreement between the GAP method and the actual birth weight using the mean percent error, mean absolute error, and mean absolute percent error. Means and 95% confidence intervals (95% CI) were calculated. Heterogeneity between studies was assessed using *I* ^2^ and tau ^2^ statistics.

**Results:**

The search identified 949 records. After a full-text review, a total of eight studies with 5,306 subjects were included. Studies were retrospective and prospective cohort studies. All studies were deemed high quality and determined to have a low risk of bias. Five studies were performed in the United States, one in Italy, one in Spain, and one in the United Kingdom. Four studies included patients with pregestational or gestational diabetes and obesity. Due to substantial heterogeneity, the random-effects model was used to estimate the effects of studies. The mean percent error was 3.1% (95% CI: 1.1–5.2), the mean absolute error was 240 g (95% CI: 205–275 g), and the mean absolute percent error was 8.0% (95% CI: 6.9–9.1).

**Conclusion:**

The GAP method of fetal weight extrapolation is an accurate approach to birth weight prediction and is suitable for use in a diverse population. The study protocol was submitted for online registration in the International Register of Prospective Systematic Reviews (PROSPERO) before the literature review was undertaken (registration number: CRD42023392977).

**Key Points:**

- Estimation of fetal weight is useful for delivery planning.
- The GAP method uses third-trimester ultrasound data.
- The GAP method is an accurate approach to birth weight prediction.

Accurate estimation of fetal weight is useful for delivery planning and labor management. Fetal weight estimates by ultrasound range in error from 6 to 15% of actual birth weight 1 2 with significantly less accuracy in predicting macrosomia and low birth weight. 3 4 With growing rates of obesity and diabetes in the population, accurate estimation is even more essential due to the increased maternal and neonatal morbidity associated with macrosomia. 5 6

From 1999 through March 2020, the prevalence of obesity among adults aged 20 and older in the United States increased from 30.5 to 41.9%, and the prevalence of class III obesity (body mass index [BMI] ≥ 40) increased from 4.7 to 9.2%. 7 The percentage of women with prepregnancy obesity rose from 26.1% in 2016 to 29.0% in 2019. 8 Similarly, preexisting and gestational diabetes has continued to rise in recent years. 9 10 This increase in obesity and diabetes is associated with an increased risk of macrosomia, 11 which decreases the accuracy of ultrasound and birth weight estimation by clinical assessment. 12 13 This creates complexities in predicting birth weight at the time of delivery.

Various methods exist to predict neonatal birth weight, including clinical assessment via Leopold maneuvers and fundal height, maternal self-estimation, and ultrasound; however, no one method has consistently performed better. 14 15 16 Additionally, clinical estimation of fetal weight may not be practical or feasible in an obese population. 17

The gestation-adjusted projection (GAP) method of fetal weight extrapolation uses third-trimester ultrasound estimated fetal weight (EFW) to predict birth weight based on the assumption that normal fetuses do not cross percentiles on growth curves. This method, proposed by Mongelli et al, assumes that fetal growth occurs linearly and suggests that the ratio between EFW at the time of ultrasound and the median fetal weight at that gestational age will be maintained until delivery. 18 This ratio can subsequently be used to predict actual birth weight based on the median birth weight at the gestational age of delivery. The original GAP method used birth weight data extrapolated from the normal reference range of Hadlock et al, 19 which was derived from 392 Caucasian women in the United States in 1991 without any fetal or maternal complications. Subsequent iterations of the GAP method utilized birth weight data from Brenner et al, 20 which was published in 1976 and was derived from 30,772 liveborn infants of different ethnicities from Cleveland, Ohio and 430 aborted fetuses from North Carolina. Application of the GAP method is achieved with the following formula 21 :



The GAP method typically uses an EFW obtained by ultrasound at 34 to 36 weeks gestation. Studies that have assessed the timing of ultrasound for utilization of the GAP method have demonstrated that ultrasounds performed at 34 to 36 weeks gestation allow for a more accurate birth weight prediction than those performed before or after this period. 3 22 One study showed that the GAP method is not influenced by extreme BMI and has high sensitivity and specificity for predicting macrosomia. 17

In this systematic review and meta-analysis, we aimed to determine the accuracy of the GAP method in predicting actual birth weight at the time of delivery. We hypothesized that the GAP method would be an accurate approach and would predict birth weight within 10%.

## Materials and Methods

### Sources

The study protocol was developed, and a systematic review of the relevant literature was completed according to the preferred reporting items for systematic reviews and meta-analyses (PRISMA) guidelines. 23 The study protocol was submitted for online registration in the International Register of Prospective Systematic Reviews (PROSPERO) before the literature review was undertaken (registration number: CRD42023392977). A literature search was performed using MEDLINE/PubMed, Embase, Scopus, and Web of Science electronic databases for studies published from database inception to June 2023. We included randomized controlled trials, cohort studies, and case-control studies of pregnant women at term gestation (≥37 weeks) with an EFW calculated by ultrasound between 34 ^0/7^ and 36 ^6/7^ weeks gestation. Studies including multiple gestations and fetuses with anomalies were excluded. A reference librarian performed the search. Keywords and search terms included “GAP,” “fetal weight,” “fetal weight extrapolation,” “ultrasound,” and “birth weight.” We reviewed the reference lists of relevant articles to isolate additional pertinent sources.

### Study Selection

All titles and abstracts were independently reviewed by two study authors (MMV and HCGN). Disagreements between the two reviewers were resolved by discussion and consensus. After the titles and abstracts were assessed, the same two authors assessed the relevant full-text articles independently for inclusion.

The quality of studies was evaluated using the Newcastle–Ottawa scale. 24 This tool assessed the quality related to the selection, comparability, and outcome of the studies. Regarding quality assessment, the tool critically evaluates such factors as cohort representativeness, ascertainment of exposure, comparability of cohorts, assessment of outcome, and adequacy of follow-up. The included studies were deemed high quality as they were allotted the maximum number of stars for each tested category.

The risk of bias was assessed using the ROBINS-I tool. 25 This tool assesses for multiple domains of bias, including preintervention, at-intervention, and postintervention. The included studies were all determined to have a low risk of bias across all evaluated domains.

Data collected from the studies included lead author, year of publication, country, patient characteristics (age, BMI, parity status, gestational age at delivery, and birth weight), sample size, and outcomes including mean percent error, mean absolute error, and mean absolute percent error, when provided. The mean percent error is the percentage difference between actual birth weight and predicted birth weight with the possibility of negative and positive values offsetting one another. The mean absolute error and the mean absolute percent error measure the absolute difference. Data on all variables was not required for study inclusion.

### Statistical Analysis

The meta-analysis evaluated the agreement between the GAP method for birth weight prediction and the actual birth weight. Untransformed means (MRAW) were used to estimate the confidence interval (95% CI) for individual studies. Heterogeneity was tested by using the *I* ^2^ statistic ( *I* ^2^ ≤ 25% defines low heterogeneity; 25% < *I* ^2^ < 75% moderate heterogeneity; and *I* ^2^ ≥ 75% substantial heterogeneity), and tau2 statistics were calculated to estimate the amount of heterogeneity of effect sizes across studies. The significance level was defined as *p* < 0.05. Higher *I* ^2^ values and statistically significant tau2 values indicate considerable heterogeneity and higher variability of effect sizes across studies. Based on the results of heterogeneity tests, the random-effects model (giving equal credit to all studies with large or small sample sizes) was used to estimate the effects of studies.

Forest plots were used to visualize the meta-analysis results. The plots display lead author names, means, standard deviations (SD), and 95% confidence intervals for all studies. In addition, they present the random-effects model, heterogeneity tests, and *p* -value, as well as the summary mean and associated 95% CI from the meta-analysis.

Funnel plots were constructed to identify the presence of heterogeneity and certain forms of publication bias. Egger's test was additionally used to test funnel plot asymmetry.

## Results

**Fig. 1:**
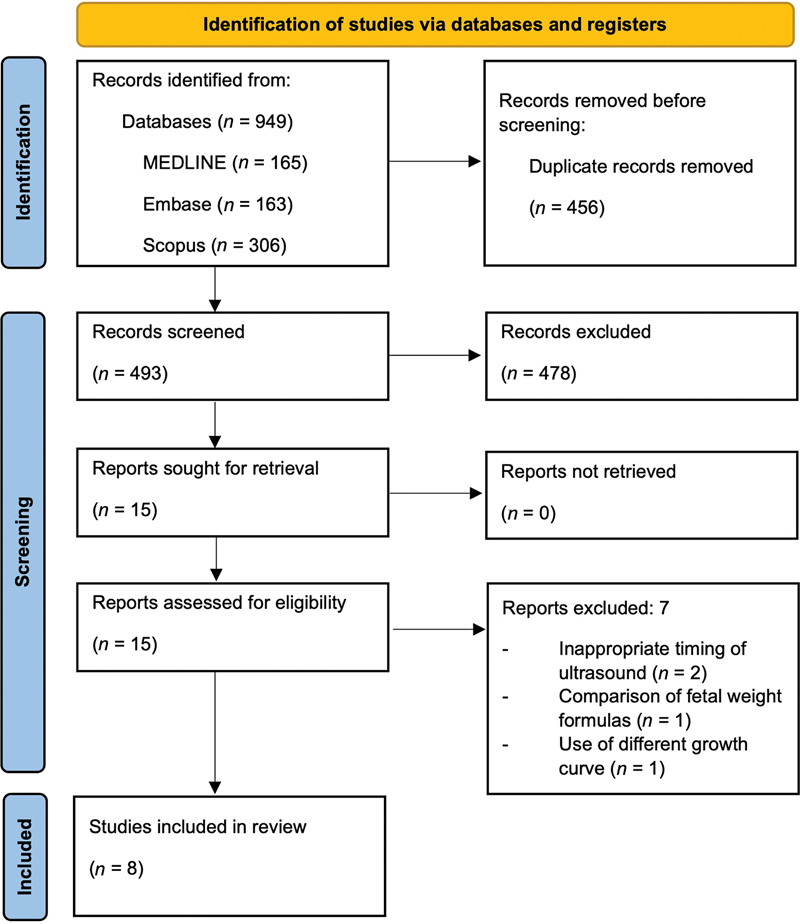
PRISMA flow diagram of retrieved studies in meta-analysis. PRISMA, preferred reporting items for systematic reviews and meta-analyses.

The initial search produced 949 articles, of which 456 were duplicates. Subsequent screening eliminated 478 irrelevant studies, yielding 15 studies for full-text review. After a full-text review, eight studies were included in the final analysis ( Fig. 1 ). 3 6 18 21 26 27 28 29 Seven studies were excluded for inappropriate timing of third-trimester ultrasound ( *n* = 2), comparison performed on fetal weight formulas ( *n* = 1), use of different growth curves ( *n* = 1), and inclusion of results that were not comparable ( *n* = 3). All studies were retrospective or prospective cohort studies. No randomized controlled trials were identified. A total of 5,306 subjects met the inclusion criteria for the studies, and sample sizes ranged from 115 to 1,823. These studies were published between 1996 and 2019. Five studies were performed in the United States, one in Italy, one in Spain, and one in the United Kingdom. Four studies included subjects with pregestational or gestational diabetes and obesity. The characteristics of included participants and studies, respectively, are detailed in Tables 1 and 2 .

**Table 1:** Baseline characteristics of study participants

	Study	Year	Age (y)	Parity (%)	Gestational age at delivery (wk)	BMI	Birth weight (g)
1	Mongelli et al 18	1996	Not reported	Not reported	39.6 ± 1.7	Not reported	3,394 ± 553.8
2	Pressman et al 3	2000	27 (14–42)	Not reported	39.3 (37.1–42.4)	Not reported	3,182 (2,097–4,481)
3	Best et al 26	2002	Not reported	Not reported	38.9 ± 1.7	Not reported	3,242 ± 550
			Not reported	Not reported	38.2 ± 1.5	Not reported	3,660 ± 707
4	Thornburg et al 27	2008	26.7 ± 6.9	42 nulliparous	38.9 ± 1.6	22.7 ± 3.4	3,202 ± 506
			27.5 ± 6.3	35 nulliparous	38.9 ± 1.8	32.4 ± 1.4	3,436 ± 664
			27.3 ± 5.5	31 nulliparous	38.9 ± 1.7	37.5 ± 1.5	3,454 ± 613
			27.5 ± 5.0	22 nulliparous	39.0 ± 1.7	45.7 ± 4.5	3,610 ± 700
5	Pagani et al 28	2014	33.8 ± 4.7	Not reported	39.1 ± 1.2	26.6 ± 5.4	3,521 ± 536
6	Schwartz et al 21	2016	33.9 ± 4.4	57 nulliparous	38.9 ± 0.8	26 ± 6	3,356 ± 438
7	Tuuli et al 6	2016	29.4 ± 6.1	36 nulliparous	39.5 ± 0.8	34.6 ± 9.5	Not reported
8	Vila-Candel et al 29	2019	31.0 ± 6.0	48.6 nulliparous	39.14 ± 1.5	12.3% with BMI ≥ 30	3,254 ± 448.4

Abbreviation: BMI, body mass index.

Note: Data are presented as mean (range), mean ± standard deviation, or proportion.

**Table 2:** Study characteristics

	Study	Year	Study design	Country	Sample size
1	Mongelli et al 18	1996	Prospective cohort study	UK	262
2	Pressman et al 3	2000	Retrospective cohort study	USA	138
3	Best et al 26	2002	Retrospective cohort study	USA	1,823
4	Thornburg et al 27	2008	Retrospective cohort study	USA	1,382
5	Pagani et al 28	2014	Prospective cohort study	Italy	125
6	Schwartz et al 21	2016	Retrospective cohort study	USA	237
7	Tuuli et al 6	2016	Prospective cohort study	USA	115
8	Vila-Candel et al 29	2019	Prospective cohort study	Spain	1,224

Participants in all studies were of similar age and at similar gestational ages at the time of delivery. The mean age ranged from 26.7 to 33.9 years. The mean gestational age at delivery ranged from 38.2 to 39.6 weeks. The BMI differed substantially among subjects with the mean BMI ranging from 22.7 to 45.7.

Comparisons were made between the EFW and actual birth weight using the mean percent error, mean absolute error, and mean absolute percent error; however, not all studies reported the same outcome statistics. When meta-analysis was performed, the overall mean percent error was 3.1% (95% CI: 1.1–5.2). The overall mean absolute error was 240 g (95% CI: 205–275 g). The overall mean absolute percent error was 8.0% (95% CI: 6.9–9.1). This degree of error would correspond to an actual birth weight between 3,220 and 3,780 g for a GAP-derived EFW of 3,500 g.

**Fig. 2:**
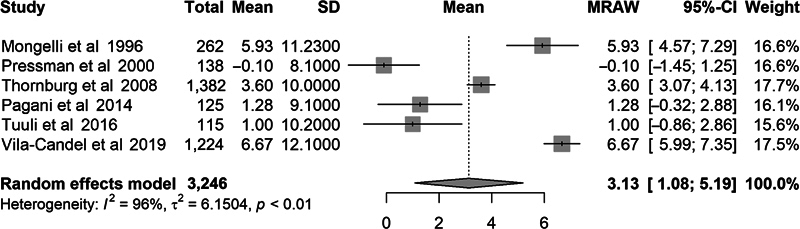
Forest plot for percent error.

**Fig. 3:**
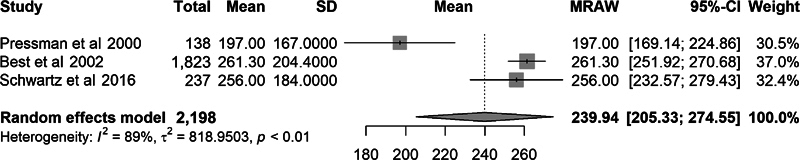
Forest plot for absolute error.

**Fig. 4:**
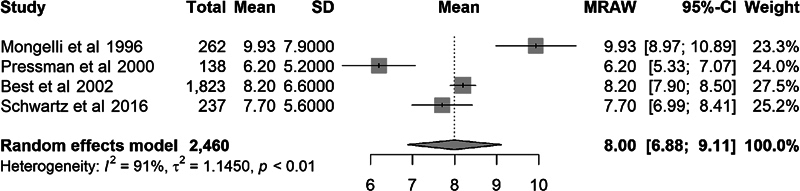
Forest plot for absolute percent error.

Due to the variation in characteristics of research subjects across studies and the large degree of heterogeneity as evidenced by an *I* ^2^ of 89 to 96%, we calculated effect size using the random-effects model. Forest plots of these outcome measures are demonstrated in Figs. 2 to 4 . The results of the meta-analysis are summarized in Table 3 . Egger's test for publication bias indicated no evidence of publication bias with *p* -values of 0.39 for mean percent error, 0.43 for mean absolute error, and 0.85 for mean absolute percent error.

**Table 3:** Percent error, absolute error, absolute percent error, and 95% confidence intervals for included studies

Study	Year	Percent error (%)	95% CI	Absolute error (g)	95% CI	Absolute percent error (%)	95% CI	Sample size
Mongelli et al 18	1996	5.93	(4.57, 7.29)			9.93	(8.97, 10.89)	262
Pressman et al 3	2000	−0.10	(−1.45, 1.25)	197.00	(169.14, 224.86)	6.20	(5.33, 7.07)	138
Best et al 26	2002			261.30	(251.92, 270.68)	8.20	(7.90, 8.50)	1,823
Thornburg et al 27	2008	3.60	(3.07, 4.13)					1,382
Pagani et al 28	2014	1.28	(−0.32, 2.88)					125
Schwartz et al 21	2016			256.00	(232.57, 279.43)	7.70	(6.99, 8.41)	237
Tuuli et al 6	2016	1.00	(−0.86, 2.86)					115
Vila-Candel et al 29	2019	6.67	(5.99, 7.35)					1,224
Overall mean		3.13	(1.08, 5.19)	239.94	(205.33, 274.55)	8.00	(6.88, 9.11)	

## Discussion

In this systematic review and meta-analysis, we demonstrated that in a diverse population, the GAP method of fetal weight extrapolation appears to be an accurate approach to estimating birth weight.

The outcomes used in this study (percent error, absolute error, and absolute percent error) are valuable measures of accuracy because the GAP birth weight is directly compared with the standard of actual birth weight. By using the mean absolute errors, studies can minimize the possibility of overestimation or underestimation, confounding the results. Our results were consistent with the hypothesis that the GAP method would accurately predict birth weight and was within 10% of actual birth weight among studies where percent and absolute percent errors were reported.

This systematic review and meta-analysis has multiple strengths. The study design, including search strategies and study selection, was predetermined in a review protocol. All included studies were noted to be of high quality and have a low risk of bias. Furthermore, this meta-analysis included a diverse sample of subjects, such as those with pregestational diabetes, gestational diabetes, and obesity.

Potential limitations include the absence of uniform reporting of outcomes across all studies (percent, absolute, and absolute percent errors) and the lack of randomized controlled trials. There was also a substantial degree of statistical heterogeneity among the included studies. Due to this heterogeneity and the diversity of the study populations, we utilized a random-effects model for the meta-analysis to minimize the effect of differing sample sizes. Additionally, due to varying study methodologies and limited access to stratified data, we were unable to assess the performance of the GAP method specifically in cases of macrosomia or growth restriction.

This study assessed the accuracy of the GAP method, and we did not evaluate its effect on maternal and neonatal outcomes. A potential area for future research is to examine whether maternal and neonatal birth injuries or complications could be avoided with improved accuracy in the detection of macrosomia. In a large retrospective cohort study of 35,548 pregnancies, macrosomia was associated with cesarean delivery for failure to progress, severe postpartum hemorrhage, obstetric anal sphincter injury, shoulder dystocia, obstetric brachial plexus injury, birth fractures, and hypoxic-ischemic encephalopathy. 30 If the GAP method does improve the prediction of macrosomia, there is the potential to diagnose and effectively counsel women on the risks of delivering a large-for-gestational-age infant.

The original GAP model was based on the assumption that fetal growth occurs in a linear fashion. 31 More recent evidence demonstrates that fetal growth in the third trimester can be influenced by several factors, including maternal nutrition, genetics, and environmental exposures. One area of future study is to assess the accuracy of the GAP method utilizing a fetal growth nomogram based on local population data or a fetal growth curve that is more representative of current understanding.

We also believe that the GAP method could be useful in low-resource settings where ultrasound may not be readily available at the time of delivery or in settings where there is less expertise or less training in obtaining biometry measurements by ultrasound. Although there are several alternative methods of birth weight prediction, in this systematic review and meta-analysis, we have demonstrated that the GAP method was a consistently accurate approach.
